# #Healthy: smart digital food safety and nutrition communication strategies—a critical commentary

**DOI:** 10.1038/s41538-020-00074-z

**Published:** 2020-10-01

**Authors:** Julie L. Schiro, Liran Christine Shan, Mimi Tatlow-Golden, Chenguang Li, Patrick Wall

**Affiliations:** 1 School of Business, University College Dublin, Belfield, Dublin 4, Ireland; 2 School of Public Health, Physiotherapy and Sports Science, University College Dublin, Belfield, Dublin 4, Ireland; 3 Faculty of Wellbeing, Education and Language Studies, The Open University, Stuart Hall Building, 2nd floor, Walton Hall, Milton Keynes, MK7 6AA UK; 4 School of Agriculture and Food Science, University College Dublin, Belfield, Dublin 4, Ireland

**Keywords:** Education, Science, technology and society

## Abstract

This paper explores how food safety and nutrition organisations can harness the power of search engines, games, apps, social media, and digital analytics tools to craft broad-reaching and engaging digital communications. We start with search engines, showing how organisations can identify popular food safety and nutrition queries, facilitating the creation of timely and in-demand content. To ensure this content is discoverable by search engines, we cover several non-technical aspects of search engine optimisation (SEO). We next explore the potential of games, apps, social media, and going viral for reaching and engaging the public, and how digital data-based tools can be used to optimise communications. Throughout, we draw on examples not only from Europe and North America, but also China. While we are enthusiastic about the benefits of digital communications, we recognise that they are not without their drawbacks and challenges. To help organisations evaluate whether a given digital approach is appropriate for their objectives, we end each section with a discussion of limitations. We conclude with a discussion of General Data Protection Regulation (GDPR) and the practical, philosophical, and policy challenges associated with communicating food safety and nutrition information digitally.

## Introduction

Traditionally, authorities and experts communicate the latest food safety and nutrition information to the public through tightly controlled mass media channels, treating the public as passive information receivers^1^. A problem with this approach is that it often fails to engage and thus educate the public^1,2^. In the past decade, the rise of search engines, smartphones, and social media have revolutionised the way people communicate, not only with each other but also with organisations. No longer confined to a passive role, the public can respond to communications in real-time by searching online or engaging on social media. This is good for communication effectiveness. The more people interact with a message, the more memorable it becomes^3^. While many commercial brands have embraced digital platforms to promote unhealthy food and drinks^4–6^, food safety and nutrition organisations have lagged behind^7^. Organisations that do utilise digital platforms often fail to maximise the platforms’ potential, instead treating them like their traditional counterparts: as one-way messaging channels^8^.

In the private sector, digital advertising spend will eclipse traditional advertising spend in the next 5 years^9,10^. Given the power and prevalence of digital communications, it is important for non-profits, government agencies, and educational institutions to have a voice online, and understand how to optimise that voice with precision targeting, A/B testing, and analytics tools. We have structured this paper to show how search engines, games, apps, social media, virality, and digital data-based tools can be leveraged at various stages of the communication process (e.g., content development, dissemination, evaluation) to improve the reach and engagement of food safety and nutrition communications. Though not a comprehensive list of digital approaches, we chose these due to their enormous potential for reaching the masses^11,12^. Throughout the paper, we draw on examples not only from Europe and North America, but also from China. We also discuss some digital examples outside the food domain, as food-related examples are often sparse. We end with a discussion of the legal and practical challenges of communicating food safety and nutrition through digital platforms.

While we are enthusiastic about the benefits of digital approaches to communicating food safety and nutrition information, we recognise that they are not without their drawbacks and challenges. To help organisations evaluate whether a given digital approach is appropriate for their objectives, we end each section with a discussion of limitations. Our hope is that by doing so, organisations will be well-equipped to implement these approaches with realistic expectations and a keen eye for challenges.

## Search engines

### Appealing to common search queries

People often use search engines such as Google to search for information on food safety and nutrition^1,13^. They also search in response to television and poster ads^14,15^. In one study, Google found that 2/3 of smartphone users report using their phone to search for more information after seeing a television advertisement^14^. By knowing what people search, organisations can tailor content accordingly, increasing the odds of reaching interested audiences with important information.

Searches primarily fall under four goals: to know, go, do, or buy^16^. Know-type queries such as ‘safe cooking temperature for chicken?’ represent a particularly important opportunity for food safety and nutrition organisations. Free platforms such as Google Trends and Answer the Public allow organisations to identify the most searched topics. For instance, if we enter the keywords ‘safe cooking temperatures’ into Answer the Public, we see that pork, chicken, and olive oil are among the most searched food items in relation to this query. Once important ‘know’ queries are identified, organisations can create communications that directly address these ‘I-want-to-know’ moments, knowing there is already a built-in audience.

### Non-technical search engine optimisation

The next step is to ensure that this content, likely hosted on the organisation’s website, is discoverable by the general public using search engines (e.g., Google). This is done through search engine optimisation (SEO). While a detailed treatment of SEO is outside the scope of this paper, we describe several tactics that require little to no technical expertise but have a substantial impact on SEO nonetheless.

The first tactic is to ensure that the title of the content is phrased as an answer to a query, e.g., ‘how to tell if you have food poisoning’. The title should use non-technical language and simple syntax, which reflects how people search^17^. This helps search engines and searchers, alike, understand the nature of your content and its relevancy to the query. The second tactic is to ensure that the content is comprehensive. For example, consider the query ‘safe cooking temperature for chicken’. A comprehensive article would not only answer this query but also anticipate and answer related queries that the searcher might have, such as how long chicken lasts in the fridge or freezer. Such comprehensiveness signals authority on a given topic (in this case, safe poultry consumption) to search engines, which search engines reward with better rankings^17,18^. While comprehensive content is typically long (>1000 words), length alone is not sufficient to ensure better rankings. Length appears to matter because it correlates with comprehensiveness, but it is comprehensiveness and not length that ultimately matters for better rankings^19^.

The second tactic is to ensure that visitors from search engines stay and engage with the content. After clicking on a search engine result, most people spend only 10–20 s evaluating the content and deciding whether to stay^20^. If the content does not clearly demonstrate its value in this timeframe, people tend to leave^20^. This matters for SEO. Search engines give better rankings to pages with better retention metrics (e.g., lower bounce rates, longer dwell times). How can organisations improve these metrics? One strategy is to make content easy to scan, making it easy to evaluate in 20 s or less. This involves using bulleted lists, beginning with the conclusion, employing descriptive and plentiful subheadings, and reducing conventional word counts by half or more^20–22^. If the content involves statistics or processes, infographics become useful. Infographics combine text, data, illustrations, and images into a single graphical narrative. Done well, they allow viewers to identify patterns and processes easier than if the same information were conveyed only through text^23–25^. Infographics, and the use of multimedia more generally, can further improve retention metrics by engaging visitors for longer^26^. Video seems to be an especially engaging format. In one study of 100 pieces of content, visitors stayed 2.6× longer on content with video than on content without^27^.

By making content comprehensive, easy to scan, and engaging, it tends to perform well on another fundamental SEO metric: backlinks (links from other websites)^28^. Backlinks improve SEO, assuming they are genuine ‘endorsements’ from reputable sites. Search engines employ many additional tactics to evaluate SEO. For example, pages with better usability (e.g., faster load speeds, responsive design) and optimised meta-data tend to rank higher on search engines^29,30^. For further comprehensive study of this topic, we refer the reader to several excellent free guides for beginners^29,30^. Table 1 summarises the non-technical SEO tactics described in this section and acts as a checklist for organisations.

**Table 1:** Non-technical SEO checklist.

Category	Why it matters for SEO
Title	
– Non-technical language	Reflects how people search; signals relevance to search engines and searchers
– Simple syntax	
– Phrased as an answer to a query	
Content	
– Comprehensive	Signals authority and quality to search engines; can increase retention metrics and encourage backlinks
– Scannable	
– Multimedia	

Food safety and nutrition organisations should also consider creating or revising content on web-based encyclopaedia (e.g., Wikipedia). Wikipedia is the fifth most visited webpage in the world as of January 2020^12^, and its pages tend to rank highly on search engines. The public perceives these sources as useful and credible sources of food safety information^1,31^. However, a study shows that Wikipedia articles tend to cite news articles rather than more authoritative sources, such as the US Food and Drug Administration^32^. Accordingly, organisations should directly contribute to Wikipedia to promote accurate and reliable information.

Limitations: By creating digital content catered to common search queries, food safety and nutrition organisations can reach audiences who are more willing and able to receive their message than other audiences, such as those watching television. A trade-off, however, is reach. Reach will be restricted to those searching specific keywords rather than the general public. Given that reach generally correlates with campaign effectiveness, creating content targeting search engines should be part of a broader communications strategy^33,34^.

## Games and apps

In recent years, game/quiz-based learning has been increasingly used to engage children and young people with food safety and nutrition information^35^. For example, the US Department of Agriculture generated two small online games (i.e., MyPlate Blast Off, and Track and Field Fuel-Up) to educate students about nutrition^36^. Food hygiene online educational games have proven successful in improving children’s knowledge and retention compared to traditional learning methods^35,37^. The application of online games/quizzes has expanded to the mobile world, and the target audience is no longer restricted to children. For instance, ‘JustFoodFun’ is a smartphone quiz on food literacy^38^. In China’s 2019 Food Safety Publicity Week, the government launched a smartphone quiz-game in collaboration with Alibaba testing players of all ages on topics of food safety, nutrition, and food science and technologies, resulting in 1.7 billion participation times within just one week^11^. This quiz game was especially successful due to heavy promotion and monetary and social incentives to play.

Nutrition-related smartphone apps have also gained popularity^39^. These apps serve a multitude of functions, from helping users to track their weight and calorie intake, to offering nutritional advice, to aiding shoppers in the interpretation of food labels^39–42^. The French app ‘Yuka’ enables users to scan product barcodes and see a consumer-friendly interpretation of the information (i.e., nutrition quality, manufacturing methods, and alerts to the presence of potentially harmful additives), and to compare product information side by side^42^. The app also crowdsources content; users can add to the database if a product is not yet included. Crowdsourcing increases engagement and reduces some burden on developers in terms of collecting raw data (i.e., information printed on food packaging)^42^.

Limitations: While some intervention studies in school settings have shown positive effects using games in food safety and nutrition education^35,37^, there is a lack of comprehensive evaluation of its impact in adult education. In other areas (e.g., health and well-being), the impact of games is mixed: 59% of the studies reported positive effects, while 41% reported mixed or neutral effects^43^. Similarly, in relation to smartphone apps, little research has examined their effectiveness (i.e., the degree of users’ engagement with the app in the real world or whether engagement with the app results in a measurable behaviour change among active users)^44^.

Nonetheless, well-designed games and apps deliver enjoyable and engaging experiences, and are likely to stimulate positive impact^35,37,43^. To gain such benefits, however, food safety and nutrition organisations must first consider the potential high cost of production and design complexity, promotions and/or the provision of incentives/rewards to encourage participation or usage^11^. Furthermore, if the app involves interactive features, such as two-way communication and open databases (which allow users to update or add information), it can be challenging to manage and process this data at scale^42^.

It can also be difficult to design educational games with high replay value. However, we suggest that this is not, in fact, a limitation. In the marketing literature, a robust finding is that increasing penetration (reaching new buyers) matters more than increasing loyalty^33,34^. Firms that focus on penetration enjoy greater market share, profits, and growth than their loyalty-focused counterparts. The theoretical reasons for this are beyond the scope of this paper, but there is little reason to expect something different for games. Games that focus on recruiting new players (i.e., penetration) over replay value (i.e., loyalty) should have greater impact at the population level simply because they reach more people. However, this is ultimately an empirical question.

## Social media

We define social media as any internet-based platform for users to post content, react to content, and build social communities. Examples include YouTube, Instagram, Facebook, Twitter, and TikTok. Messaging apps such as WhatsApp and WeChat exemplify this definition less so, though are often considered ‘social media’ by the industry^12^. We thus consider them as such, though our focus is on the more “full service” platforms.

A top objective of organisations using social media is to expand the reach of their communications^45^. To achieve this objective, we first need to understand how social media works. Most social media platforms filter content through an algorithm, showing only the ‘best’ content to users. Algorithms determine the ‘best’ content by analysing the level of engagement (e.g., likes, shares, comments) on posts individually, and the account overall. Organisations that prioritise post quantity over post quality can unknowingly hurt their reach by posting subpar content that gets little engagement^46^. By posting better content less often, overall engagement metrics should improve, triggering the algorithm to show the organisation’s posts more often and to more users^46^. But even for accounts with high engagement rates, the reach of their unpaid posts is often limited by the size of their fan base unless organisations can enliven fans to share the posts with friends. If so, organisations can increase the reach of their content manyfold^47^.

How can organisations enliven their fan base to like, comment, and share content on social media? One way is by producing viral-worthy content (see ‘Optimising content’). Another is by motivating user-generated content (UGC), thereby reaching friends of fans. Research suggests that the highest quality UGC stems from users motivated by social or intrinsic rewards (e.g., social recognition, self-actualisation), not financial ones^48^. Organisations should make social recognition a major part of UGC campaigns by reposting and promoting user submissions and encouraging users to interact with each other. In a competition to raise awareness of food safety, the city of Hangzhou (southeast China) called for user-generated essays on their WeChat account, then judged winners from 300+ submissions by the number of views and likes. The most popular essay reached nearly 13,000 views and 6000 likes during the first 3 days of posting—a much higher level of public engagement compared to the organisation’s other campaigns^49^. UGC can also be a launchpad for virality. UK chef Jamie Oliver’s #AdEnough campaign called for better regulation of junk food ads seen by children. The public was urged to post images of themselves hiding their eyes on social media^50^. This campaign generated substantial activity extending to mainstream media^51^.

Organisations may wonder why they should invest in encouraging UGC when they could just divert those resources to paying for reach, if that is the ultimate goal. The reason is that the payoff of a successful UGC campaign is greater than reach alone. When an organisation’s message is delivered by a friend, the message has more impact^52,53^. The influence of friends and family on social media may be especially prominent in countries with a collectivistic culture, such as China. In a cross-cultural study of social media, Chinese users kept tighter social networks and placed more trust in these networks than users from an individualistic culture (the United States)^54^. Another study found that in China, people consider friends and relatives trustworthy sources of information on food safety and nutrition^55^. Together, this suggests that UGC may be particularly influential in collectivistic cultures, especially China. Interestingly, UGC can also have a persuasive influence over those making the content because the creative process leads to greater elaboration of the message^56^. Hence, with UGC, organisations have the potential to increase both the reach and persuasiveness of their communications.

Other ways to increase engagement on social media include using video content. In a study of 777 million Facebook posts, posts with video garnered 59% more engagement than other types of content. Posing questions came in second, and posting photos came in third for engagement^57^. Another study, conducted by Twitter, found that Tweets with video garnered 10× the engagement of Tweets without video^58^. Similar findings have been observed for Instagram^59^ and LinkedIn^60^. Given the engaging nature of video content, it is important to know how to optimise this format for social media. A critical point to highlight here is that over 80% of people watch social media videos on mute^61^. People frequently view videos in situations where sound would be disruptive, and much of the video content on social media is muted by default. To compete in this environment, food safety and nutrition organisations should ensure their videos are understandable and enjoyable without sound. Captions, especially embedded captions (which cannot be turned off), should improve a video’s propensity for impact simply by making it more conducive to how social media users consume content. Captions are also an important part of making web content more accessible^62^.

Using hashtags can trigger engagement further. For example, on Twitter, Tweets with hashtags received twice the engagement of Tweets without hashtags^63^. Using hashtags popular with the audience can increase the discoverability and reach of content^63^. Further, content should be tailored to the characteristics of the audience. In one study of Facebook ads, content that was tailored based on users’ Big 5 personality characteristics (estimated based on users’ ‘likes’ of pages and posts) received more engagement than untailored content^64^. Many free tools exist to help organisations understand the demographics and psychographics of their fan base so they can craft content that resonates with their audiences, including tools built into the platforms themselves (e.g., Facebook Audience Insights, Twitter Analytics, etc.)^65,66^.

Finally, organisations should remember that social media platforms are two-way communication channels and should be treated as such; doing so can make social-media-based health interventions more effective. By posing and answering questions, engaging with users’ posts, encouraging engagement between users, and motivating UGC, organisations can foster a sense of community on their social media page^67,68^. This can positively impact users’ perceptions of social norms, self-efficacy, and social support, increasing the likelihood that a given social-media-based health intervention is successful^67,69,70^.

Table 2 lists the most-used social media platforms worldwide and their corresponding demographic profiles. The demographic profiles are based on US data only, but globally the trend is similar: social media skews younger^71^. TikTok is particularly notable due to its rapid growth since 2018 when it first debuted outside China^72^. While TikTok is designed for short-form mobile videos^73^, the app has clear educational potential. In 2019, ‘knowledge-based content videos’ were shared 14.89 million times on the app^72^. The World Health Organisation has been using TikTok to share information about COVID-19 to its 2.6 million followers. But some of the major food safety and nutrition organisations (e.g., the US Food and Drug Administration, the European Food Safety Authority) do not have TikTok accounts at the writing of this paper.

**Table 2:** Social media penetration and usage statistics^152,153^.

Platform	Global monthly penetration (in millions)^a^	% Using platform daily (US Only)^b^	% Using platform by demographic (US Only)^b^					
			Overall	18–24	25–29	30–49	50–64	65+
Facebook	2603	74%	69%	76%	84%	79%	68%	46%
YouTube^c^	2000	51%	73%	90%	93%	87%	70%	38%
WhatsApp	2000	–	20%	20%	28%	31%	16%	3%
FB Messenger^c^	1300	–	–	–	–	–	–	–
WeChat	1203	–	–	–	–	–	–	–
Instagram^d^	1082	63%	37%	75%	57%	47%	23%	8%
Douyin/TikTok	800	–	–	–	–	–	–	–
QQ	694	–	–	–	–	–	–	–
Sina Weibo	550	–	–	–	–	–	–	–
QZone	517	–	–	–	–	–	–	–
Reddit	430	–	11%	21%	23%	14%	6%	1%
Kuaishou	400	–	–	–	–	–	–	–
Snapchat^d^	397	61%	24%	73%	47%	25%	9%	3%
Pinterest	367	–	28%	38%	28%	35%	27%	15%
Twitter^d^	326	42%	22%	44%	31%	26%	17%	7%
LinkedIn	–	–	27%	17%	44%	37%	24%	11%

– Indicates data not collected.

^a^Sourced from Statista [152].

^b^Sourced from Pew Research Center [153].

^c^Global monthly penetration data may be underestimated; data unchanged in the last 12 months.

^d^Platform does not publish monthly average user (MAU) data; Statista sourced data from third-party sources.

Limitations: An overview of the advantages, drawbacks, and best practice of social media in food safety and nutrition communication can be found in the literature^1,74–76^; however, some scholars argue that evidence-based guidelines and controlled studies of impact are still very limited^76^. Further, since social media skews young, traditional media channels such as television are better equipped for reaching all-age mass audiences^33^.

It can also be challenging to stimulate UGC, and a successful UGC campaign often requires an upfront investment in promotion. When Pepsi wanted to create a Super Bowl commercial that comprised UGC, they used influencers, celebrities, television, 350 outdoor media placements, and a Times Square media takeover. Given this substantial investment, Pepsi unsurprisingly had one of the most successful UGC campaigns of the 2013 Super Bowl^77^. While Pepsi is an extreme example, a successful UGC campaign typically needs some initial promotion to spark engagement.

## Going viral

### Optimising content

Viral content is content that has achieved extensive reach through voluntary sharing by individuals and the media. While there is no rote formula for going viral, three strategies can increase the chances: making content that evokes strong positive emotions, making content that is particularly useful at a glance, and seeding content with influential sources. In this section, we focus on the two content-based strategies.

Emotion plays a major role in virality. Specifically, positive content tends to be more viral than negative content^78–82^. However, valence is not the only factor. Arousal matters as well. Content that evokes high arousal (e.g., anger, anxiety) is more viral than content that evokes low arousal (e.g., sadness)^78,81,83,84^. This helps explain why food safety rumours usually spread faster than reliable information because rumours are usually written in a way that triggers anger and anxiety.

Among the positive emotions, humour is a particularly useful viral strategy^85–87^ and one that has been successfully implemented by several food safety and nutrition organisations. One example is ‘The Real Bears’ anti-soft-drink campaign, which parodies an ad from Coca-Cola where polar bears joyfully drink Coke. In the parody, the polar bears suffer real-life health consequences of drinking soda (e.g., tooth decay, obesity, type 2 diabetes)^88^. The video received over 2.7 million views on YouTube, coverage from American mainstream media (including USA Today and AdWeek), and international coverage as well^89–92^. While there is some concern that humour can undermine serious messages, the increased exposure attributable to humour may outweigh this risk^93^.

Beyond emotion, content that is practically useful and packaged in an easy to digest format can also increase the virality of content^78^. The ‘Know Your Lemons’ campaign taught the signs of breast cancer through a visual of 12 deformed lemons. The image has been viewed over 200 million times and shared by at least 36 media outlets^94^, not only because it was useful but also because it was easy to understand at a glance^95^. Useful, easy to digest visuals seem particularly apt for the food safety and nutrition domain where education is a major objective.

Limitations: Viral marketing is a high-risk, high-reward strategy. While the content itself is critical for success, the seeding strategy can be more critical still (see ‘Seeding for virality’). When successful, viral marketing can be more cost-effective than traditional media, but not necessarily if the seeding strategy involves buying traditional media, which is often the case^33^. Further, it is difficult to craft viral content in a way that balances entertainment and the message, itself. One paper found that advertisement persuasiveness dropped by 10% for every increase in one million views because virality correlates with more entertaining (e.g., humourous) and less informational elements^87^. The drop in persuasiveness can be offset by the increase in reach, but only up to a point. The paper estimated, based on a simulation, that after three to four million views, reach ceases to positively offset the decrease in persuasiveness. While some ads succeeded in being both informative, entertaining, and viral, these instances were rare. In other words, virality is hard and valuable virality is harder^85^.

However, even if content fails to trigger sharing and media coverage on a national or even international scale, it can still have impact. If content is designed to evoke strong positive emotions or be particularly useful at a glance, it will be well-poised to generate reach and engagement across social media, albeit on a smaller and more localised scale^78,96^.

### Seeding for virality

Being positive and useful is rarely enough for virality. The main determinant for whether a piece of content goes viral is whether an influential source (e.g., an influencer, celebrity, or the media) shares the content, thereby exposing it widely^97^. Organisations can thus increase their odds of going viral by ‘seeding’ their content with influential sources. For example, the UK-based Food Foundation recently launched the ‘Veg Power’ campaign in collaboration with two celebrity chefs and a well-known medical expert^98^. To date, the campaign has successfully gained millions-worth of free advertising to promote the eating of vegetables among children^99,100^. Another example is China’s 2017 National Nutrition Week. Six A-list celebrities were invited to promote healthy eating communications offline and online^101^.

Partnering with influencers may be the most accessible strategy because it requires no prior connections with celebrities or the media. Influencers are individuals with large followings on social media^102^. Views differ on how many followers are needed to constitute an influencer, but the minimum cutoff seems to be 1000^103^. There are several tools to help organisations identify influencers who fit their values and target audience. For example, the paid tool Klear gives detailed information on influencers’ engagement metrics and audience demographics and psychographics. Chosen influencers do not have to be ‘big’ influencers with million-level followers (who normally expect to be paid); ‘micro’ influencers (influencers with 1000–10,000 followers) can be strategic because they often have more engaged followers, in addition to being less expensive^103,104^. Even in the absence of a formal influencer strategy, engaging with influencers (e.g., by following, commenting, liking, and sharing their content) can increase the chances that an influencer will share an organisation’s content of their own volition.

Some seeding activities may require sufficient financial commitment. However, even modest budgets can accommodate a seeding strategy since influential sources may be willing to collaborate for free for a good cause^11^. Even in the case of no budget, organisations can achieve virality; according to the analytics company, WARC, campaigns that achieved virality on negligible budgets did so by successfully tapping into news, memes, and cultural trends^105^. Organisations can attempt to kickstart unpaid virality by posting on their own social media accounts (i.e., self-seeding). If the content receives an abnormally large number of likes, comments, and shares relative to what would be expected by the algorithm, the algorithm may recommend the content to new audiences (e.g., through suggested content)^106^. An influential source may then see the content and share it on their own social media account or in the media. This explains how content can go viral accidentally.

The above scenario, whereby content goes viral with the unsolicited help of social media algorithms, influencers, and/or the media, hinges on content being abnormally engaging and often, luck. Abnormal engagement is largely determined by the presence of viral-worthy characteristics (‘Optimising content’), though content format may also matter. As noted in ‘Social media’, video content is more engaging than any other type on social media. Thus, viral strategies hoping to rely on the unsolicited help of social media algorithms (which recommend content based on engagement metrics) may benefit from making viral-worthy content in video form, especially if the video is uploaded directly to the platform (‘native video’) versus linked from an external source^107^. For instance, Facebook is more likely to show videos uploaded on Facebook than linked from YouTube because Facebook wants to incentivize users to host content within the Facebook ecosystem.

Limitations: Even with viral-worthy content and a team of influencers, going viral is not a guarantee. Further, while viral marketing may appear to offer high impact for little cost, seeding strategies with influencer and celebrity support often cost money, though as noted above, some may be willing to participate in a good cause for free.

## Digital data-based tools

### Algorithmic targeting

Traditionally, organisations identify their target audience(s) mainly based on socio-demographic factors, then buy media space accordingly. For instance, the UK’s Salt Awareness campaign used women’s magazines and carefully placed offline events to reach the target group—women aged 35–65 from lower-middle/working class families^108,109^. However, consumers’ likelihood of engaging with food-related communications is more influenced by those difficult-to-observe factors (e.g., knowledge level, food beliefs, food choice motives, and health conditions)^110^. Even with insights on these factors, the targeting precision of traditional media is typically too blunt to implement them^34^.

Digital targeting tools, however, greatly improve targeting precision and accuracy due to machine learning algorithms based on individuals’ browsing behaviour (e.g., clicks, likes) and social networks. One such tool is Facebook’s ‘Lookalike’ audience feature, which can identify an untapped audience that shares similar interests and behaviours to a ‘seed’ audience specified by the organisation^111^. For example, Eat Grub, a small company promoting insect-based protein for nutrition, used Facebook’s Lookalike feature to target Facebook users who resembled their existing customer-base^112^. Google, Quantcast, and IBM all offer similar ‘lookalike’ targeting options. Machine learning algorithms also make it possible to optimise targeting based on specific campaign goals, such as web traffic or call-to-actions (e.g., app installations, event sign-ups, etc.), which has proven useful in increasing campaign effectiveness^113^.

Limitations: Algorithmic targeting can be employed to expand reach to potentially interested audiences who are difficult to reach using traditional mass media channels, and to increase the persuasiveness and perceived relevance of the campaign message^64^. However, organisations should be wary of refining targeting so much that overall reach dwindles. It is critical to balance tight targeting with mass media exposure^34^. Finally, there are ethical and legal questions regarding the use of personal information on which targeting is based. We return to these in ‘Legal concerns and practical challenges’.

### A/B testing

A/B testing, also known as split testing, can help organisations optimise the messaging of their campaigns. A/B testing is the practice of disseminating two or more versions of the same content to a small randomly selected audience to determine which version performs best on a given criteria (e.g., clicks). Most advertising platforms offer A/B testing and will automate the process such that the best performing campaign is automatically identified and disseminated to the audience at large.

Headline optimisation is a major area of A/B testing and one that can have substantial impact on message engagement and reach. Top media sites spend considerable time optimising their headlines to make them stand out in the massively cluttered digital space^114^. A good headline can increase the reach of content substantially. Consider the YouTube video ‘Zach Wahls speaks about family’ (Wahls is an American social activist raised by a lesbian couple). Views of the video jumped from one million to seventeen million when a more provocative and indicative title was used: ‘two lesbians raised a baby and this is what they got’^114^.

Typically, a good headline evokes curiosity and/or strong emotions. Curiosity-evoking headlines are most appropriate for garnering views and shares on social media, while question-answering headlines are most appropriate for SEO (e.g., ‘food-poisoning diagnosis: how to know if you have it’). The WeChat account References for Food & Diet is especially good at using headlines that arouse curiosity. Below are example titles of their most shared articles: ‘What is the most toxic food that causes 10,000 deaths a year’ (food borne disease) and ‘The French Master never uses preservatives, but he can make broth that is still good after 100 years’ (pasteurisation). In addition, a good headline is also often conversational and fun, for instance ‘No, the CDC didn’t say you can’t put chickens in Halloween costumes’—an article about safe handling of chickens^115^.

Limitations: A/B testing requires computer expertise if the A/B automation programme is not embedded in the advertising platform. If food safety and nutrition organisations decide to use their own platforms for testing (e.g., official website, email lists), they need to have a reasonable level of website traffic or emails. Otherwise, it will be difficult to obtain the sample sizes required for meaningful and reliable testing. Another issue is A/B testing offers insights on metrics which may or may not covary with message effectiveness. Therefore there is a risk of being misled by ‘seemingly successful’ results (e.g., version A attracts a larger audience but not necessarily the target group, or version B’s click-through rate is higher but the viewers are actually less convinced by the message).

### Analytics tools

Traditionally, food safety and nutrition organisations use focus groups and surveys to pilot-test communications and evaluate the impact of their campaigns^116,117^. The results typically have high generalisability and reliability, though the process can be costly and slow. Website and social media monitoring can supplement traditional methods by offering spontaneous, real-time insight into the organisation’s communications so that quick optimisations can be carried out. There is a range of tools to track and optimise communications, such as Google Analytics, SimilarWeb, and SEM Rush. They allow organisations to identify which content is connecting with their audience, how people interact with a website, whether the audiences’ behaviours differ by demographic/psychographic factors, and how the website’s performance compares to other similar websites. For social media, most platforms have built-in analytics functionality (e.g., Facebook Insights, Twitter Analytics), which help organisations identify the types of content that resonate best with their audience. While this data is private to each account, online tools (e.g., Sprout Social, Keyhole) can estimate similar social media metrics for any public-facing social media account. Monitoring others’ social media accounts and websites can help organisations benchmark their performance and optimise current and future content by seeing what works well for others.

In the Food Hero Campaign, a US project helping low-income mothers to improve their children’s eating habits, the team used Google Analytics, Facebook Insights, and Pinterest Analytics to gauge what content resonated with the audience^118^. Social media analytics informed the campaign team that chicken-based recipes were the most popular ‘Food Hero’ recipe category, which they then promoted across all channels. In the UK and Ireland, food safety and nutrition organisations are actively using digital media monitoring tools to gauge public reactions to their communications and campaigns^119^.

Limitations: In 2016, Facebook acknowledged an error in its calculation of video view time, which inflated metrics, triggering a lawsuit from advertisers^120,121^. While such inaccuracies seem to be rare, it is important for organisations to recognise that they can occur. Online metrics are also limited to the online population and are especially influenced by those most active or vocal online. This makes it difficult to draw population-level conclusions. Online metrics are also often ill-suited to measure behavioural outcomes^75^. It is thus important to use online metrics in conjunction with more traditional research methods.

Further, investing in analytics tools does not guarantee campaign effectiveness. In fact, many organisations see only modest returns^122^. This is not necessarily the fault of the tools, but rather a consequence of data fragmentation, data overload, and/or lack of talent^122^. Data fragmentation occurs because multiple tools are needed to capture an organisation’s complete digital presence. Multiple tools result in multiple datasets of various coding schemes, variables, and completeness. This fragmentation, alongside the sheer volume of data available, makes it difficult to identify meaningful patterns and form a cohesive story. Even with talented analysts, clear communication objectives are needed to focus the analytics efforts. Thus, while analytics tools have great promise, their usefulness largely relies on clear direction from the organisation, knowledgeable staff, and the accuracy of the metrics, themselves^122^.

## Legal concerns and practical challenges

### Legal issues

The application of some of the strategies described above may be affected by privacy and data protection legislation and regulation in some regions. The EU’s General Data Protection Regulation (GDPR) was implemented in May 2018, and similar data protection laws may follow in other jurisdictions, for example in California, India, and Brazil^123–125^. The GDPR upholds the fundamental right of individuals in the European Union (EU) to have their personal data protected, used fairly and legally, and accessible upon their request^126^. There are six lawful reasons for processing personal data; one of these is consent (others include carrying out contracts, performing tasks in the public interest, and protecting a person’s vital interests, among others)^126^. To obtain consent, organisations that process EU data have started providing privacy notices to users. These purport to enable web users to make an informed decision about consent to processing their data. With few exceptions, however, these notices have been found to be designed to favour acceptance of data processing. These breach GDPR, as their interface is designed either to nudge users to accept the notice, or to obstruct users from rejecting it^127^. However, enforcement remains weak, and the practice is widespread.

Companies such as Facebook appear to be seeking to minimise GDPR’s impact by moving users’ data away from GDPR protection zones and prohibiting user access to the platform altogether if users do not provide consent^124,128^. However, such “consent or close your account” approaches are currently subject to major GDPR-based legal challenges in several EU countries^129,130^. Furthermore, complaints were filed in September 2018 with European data protection authorities in Ireland and the UK regarding the legality of the advertising technology ecosystem^131^, and although action remains to be taken, further European Court of Justice rulings have negated the legality of data-fuelled targeting strategies^132^. In China, the government launched a pragmatic guide for data protection in 2017—the Personal Information Security Specification; however, data protection is carefully balanced with its national ambitions for the development of artificial intelligence^133^. Most Chinese web-users are willing to trade off some degree of privacy for the convenience of web-based services^134^. Overall, it is therefore too early to draw conclusions about the impact of GDPR and other comparable data protection regulations on digital communications, privacy, data management, and consent, which is likely to play out over a number of years.

### Practical, philosophical, and policy challenges

Food safety and nutrition organisations may face challenges in harnessing digital platforms and tools. The first challenge involves resistance from stakeholders. A pan-European survey with food safety and nutrition-related stakeholders shows that some are reluctant to engage with digital media technologies because of fear of the unknown and the perception that the digital media world is evolving so fast that tools of today will be outdated tomorrow^1,8^. The second challenge is related to human resources and cost. Communicating through digital means may be less expensive than traditional mass media (e.g., television) in some cases, but it requires adequate human resources and time investment to manage campaigns^135^. Activities, such as digital training for staff and partnerships with experts can help food safety and nutrition organisations overcome these challenges.

It should be noted that digital tactics are mainly developed by the private sector for relatively straightforward goals of increasing brand awareness or selling a product/service. Food safety and nutrition communication is often more complicated than selling a brand or product, involving explanations of scientific concepts and uncertainties (e.g., controversies and conflicting evidence around a topic)^136^. In these cases, food organisations should carefully balance the use of certain viral marketing tactics (e.g., humour) with maintaining the accuracy and seriousness of the message^93^.

In relation to healthy eating, it is furthermore important to recognise that it is not possible to simply use digital platforms and tools to counter the scale of marketing that promotes unhealthy food items. For example, junk food advertisers in England spend nearly 30 times what the government spends on promoting healthy eating^137^, and marketing for fruit and vegetables represents just 2.5% of the UK’s annual food advertising spend^138^. Further, adolescents respond more positively to social media marketing for unhealthy foods—compared to healthy foods or non-food items—in terms of attention, peer ratings, likelihood to ‘share’ content, recognition, and recall^139^. Therefore, it is important not to expect public authorities to enter a marketing battle against commercial actors or to use the same evaluation scale (e.g., million-level views) to judge whether a public health campaign is successful.

Indeed, commercial advertising for unhealthy practices as well as health misinformation are both rife in digital media. Both are characterised by high-attention and high-impact features that food safety and health information may struggle to emulate. As digital platforms are optimised for high impact rather than accuracy or health, health authorities also need to advocate for removal of health misinformation and for restrictions on advertising for unhealthy practices (smoking, drinking, eating junk food). Examples of advertising restrictions are the WHO Framework Convention on Tobacco Control and its recommendations on food marketing^140–142^, and the proposed UK restrictions on online advertising of unhealthy foods^143^. Regarding health misinformation, while social media platforms can identify and remove some of it, much is still missed. Despite platforms’ attempts to rapidly remove misinformation on COVID-19^144,145^, remaining misinformation was still viewed an estimated 460 million times on Facebook in April 2020, a pivotal month in which the COVID-19 crisis intensified globally^146^. It is estimated that Facebook posts from the ten most influential websites spreading misinformation garnered nearly four times the views of reputable analogous posts from health authorities such as WHO, in part because these posts triggered abnormal engagement by being sensationalist, triggering Facebook’s algorithm to amplify the content^146^. This makes clear that countering misinformation with accurate information is not sufficient. Removing misinformation, reducing the virality of such content, and correcting it with accurate information are urgent priorities for regional and global digital regulators and the platforms themselves. Research is also needed on how to best help the public develop critical health and media literacy skills.

Finally, digital communications should complement, not replace, traditional methods. While a solely digital approach may work for younger audiences^147^, often the greatest campaign impact comes from combining traditional and digital approaches^76,148^. Using both digital and traditional channels ensures broader reach and more frequent message exposure—both of which are crucial for campaign success^34,149^. Using both also opens the potential for digital and traditional channels to amplify each other^8^. For instance, adding complementary digital content to television advertising increased return on investment by 60% in a study of over 5000 campaigns worldwide^148^. Similarly, Jamie Oliver’s ‘pink slime’ video became a social phenomenon partly because it was picked up by television and newspapers^150^ and Veg Power achieved substantial reach and awareness through its television campaigns^151^. It makes sense to utilise the broadest array of channels available, rather than only digital or only traditional ones.

## Conclusion

This paper described and critically commented on the implications of digital platforms and tools in communicating food safety and nutrition. We hope this paper sheds light on the many ways a digital approach can complement and expand traditional methods, content creation, content dissemination, and campaign optimisation.

## Data Availability

Data sharing is not applicable to this article as no datasets were generated or analysed.
